# Smart Metamaterial Based on the Simplex Tensegrity Pattern

**DOI:** 10.3390/ma11050673

**Published:** 2018-04-26

**Authors:** Anna Al Sabouni-Zawadzka, Wojciech Gilewski

**Affiliations:** Faculty of Civil Engineering, Warsaw University of Technology, 00-661 Warsaw, Poland; w.gilewski@il.pw.edu.pl

**Keywords:** metamaterial, smart material, smart structure, tensegrity

## Abstract

In the present paper, a novel cellular metamaterial that was based on a tensegrity pattern is presented. The material is constructed from supercells, each of which consists of eight 4-strut simplex modules. The proposed metamaterial exhibits some unusual properties, which are typical for smart structures. It is possible to control its mechanical characteristics by adjusting the level of self-stress or by changing the properties of structural members. A continuum model is used to identify the qualitative properties of the considered metamaterial, and to estimate how the applied self-stress and the characteristics of cables and struts affect the whole structure. The performed analyses proved that the proposed structure can be regarded as a smart metamaterial with orthotropic properties. One of its most important features are unique values of Poisson’s ratio, which can be either positive or negative, depending on the applied control parameters. Moreover, all of the mechanical characteristics of the proposed metamaterial are prone to structural control.

## 1. Introduction

Metamaterials are usually defined as man-designed and man-made, which are not observed in nature, composite structures with unusual or non-typical properties [1,2,3]. Features of metamaterials are determined mainly by morphology of the structure in the scale bigger than molecular, and in smaller degree by chemical or phase composition. This area has been under considerable and important scientific research in recent years. Many state-of the-art applications refer to electromagnetic waves and phenomena [4,5,6], solar photovoltaic cells and panels [7,8], energy absorption, including seismic [9] and acoustic waves [10,11], and mechanical metamaterials [12,13,14,15,16] (for example, with unusual dynamic properties, negative Poisson’s ratio, non-typical modulus of extension and volumetric changes, ultra-light, and ultra-stiff materials).

Smart materials are the materials that are able to convert one form of energy (mechanical, magnetic, electrical, etc.) into another in a reversible and repeatable process [17,18]. They are capable of sensing changes in the environmental conditions, responding to them in a predetermined manner, in an appropriate time and returning to their original shape as soon as the stimulus is removed.

Smart structures are the structures with the ability to sense and respond adaptively to changes in their environment [19,20]. This feature distinguishes them from the conventional ones. Whereas, the main purpose of the traditional structures is to provide strength and carry loads acting on them, the smart ones adapt in a pre-designed manner to a functional need, modifying their shape, stiffness, or damping characteristics in order to minimize the deflection and possible damage.

In the context of the above definitions, the metamaterial with smart features is more close the term smart structure than smart material.

Standard engineering materials when compressed along a particular direction are the most commonly observed to expand in the directions orthogonal to the applied load. This property is measured by a Poisson’s ratio, which is a good example to characterize the mechanical metamaterial. Positive Poisson’s ratio in the range of 0.0 to 0.5 is observed for the majority of engineering materials, which means that it is a typical material property. However, theory of elasticity permits negative values and for anisotropic materials also the coefficients bigger than 0.5. Negative Poisson’s ratios are not observed in nature and this is why metamaterials with such a property are being looked for. An interesting group of up to date efforts in this area are metamaterials that are based on the origami patterns [21,22,23,24,25,26], inspired by the art of paper folding. The most efficient is the Miura-Ori origami pattern. 

Another interesting type of structures that allow for building materials with negative Poisson’s ratios are tensegrities. The term “tensegrity” was first introduced by Buckminster Fuller (see [27] for historical details). Several definitions of this concept can be found in the literature [27,28,29]. For the purpose of this paper, a tensegrity structure is defined as a pin-jointed system with a particular configuration of cables and struts that form a statically indeterminate structure in a stable equilibrium. Tensegrities consist of a discontinuous set of compressed elements inside a continuous net of tensioned members, which have no compressive stiffness. Infinitesimal mechanisms, which occur in tensegrity structures, are balanced with self-stress states [30,31]. Occurrence of a self-stress state in a structure indicates that there is a certain set of internal forces in structural members, which are independent from external loading and boundary conditions because they are in self-equilibrium. 

To major advantages of tensegrity systems belong: large stiffness-to-mass ratio, deployability, reliability, controllability [27,28], as well as programmable deployment [32]. Moreover, tensegrities have some unique features that result from the infinitesimal mechanisms, which are stabilized by self-stress forces. It is possible to control their static and dynamic properties by adjusting the pre-stressing forces [33,34,35,36].

As it was presented in [37], there are some particular features of tensegrity structures, following which one can classify them as smart structures. There are: self-control, self-diagnosis, self-repair, and self-adjustment (active control).

Self-control of tensegrity systems consists in self-stiffening of the structures under the applied load that causes displacements consistent with the infinitesimal mechanism mode. External loading acts similarly to the self-stress—it eliminates singularity of the problem, additionally, pre-stresses the structure and stiffens it. Self-diagnosis relates to the possibility of damage detection and identification by measuring the internal forces in active members. Damage of one structural element affects the distribution and level of self-stress in the whole structure. Self-repair of tensegrity structures is realized by adjusting self-stress forces. A proper change of pre-stressing level can compensate the damaged element and restore the values of structural displacements from before damage. Self-adjustment (active control) in regard to tensegrity systems is related with the ability of self-adjustment through self-stress forces. Both the pre-stressing of the whole structure and its part causes a stiffening of the system and the reduction of its displacements. Therefore, active control of tensegrities can be realized by adjusting the level of self-stress in only one selected part of the structure.

The objective of the present paper is to develop a metamaterial based on the 4-strut simplex tensegrity module [38], which was exhibiting the smart structure features. A continuum model [39,40] is applied to identify the qualitative properties of the proposed metamaterial. Its mechanical characteristics can be controlled with the self-stress state and cable to strut properties ratio, including positive or negative values of Poisson’s ratio. According to the best knowledge of the authors, there are almost no papers in this field in the available literature, with the first valuable attempt on the mechanical response of three-dimensional (3D) tensegrity lattices by [41].

## 2. Unit Cell

**Figure 1:**
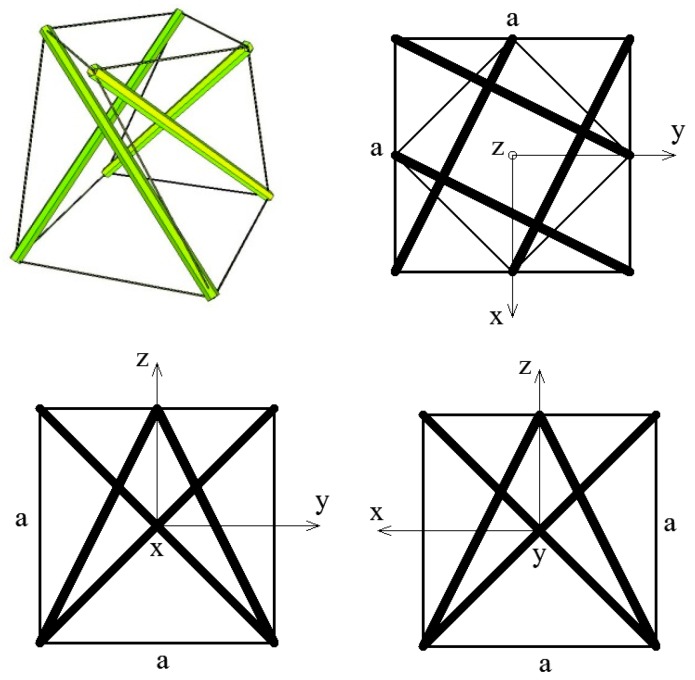
Geometry of a unit cell—a 4-strut simplex module—axonometry and three views.

The proposed metamaterial is based on one of the best known tensegrity modules—a 4-strut simplex (Figure 1). As all typical tensegrity structures, it is a pin-jointed system consisting of isolated compressed elements (four struts) inside a continuous net of tensioned members (twelve cables) [27,28,29]. The 4-strut simplex module is obtained from a regular prism by rotating one of its bases 135 degrees clockwise or counter clockwise.

**Figure 2:**
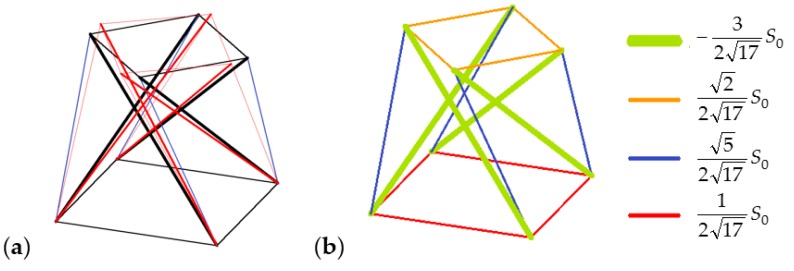
4-strut simplex: (**a**) Infinitesimal mechanism; and (**b**) self-stress state.

One of the unique features of tensegrities are infinitesimal mechanisms that are balanced with self-stress states [30,38]. The considered simplex module has one infinitesimal mechanism (Figure 2a) and one corresponding self-stress state (Figure 2b)—self-stress is expressed by the relative forces in struts and cables with a multiplier *S*_0_, which can be any positive real number.

Any material or system that is based on tensegrity is complicated regarding both its geometry and mechanics. Therefore, it is often difficult or even impossible to determine and understand its properties using typical tools. Standard methods, such as the finite element method, enable analysing any kind of structure, but they do not explain if the analysed system exhibits anisotropic, orthotropic properties, or is characterized by some other type of elastic symmetry. Using such methods, it is also hard to describe unusual properties in the sense of a metamaterial.

In order to analyse all of the untypical, unique features of tensegrity systems and to identify their properties, a continuum model [39,40] is suggested. The aim of the proposed model is to facilitate the identification and the understanding of mechanical characteristics of tensegrities through the qualitative comparison with a continuum body [42,43] with equivalent features. The model is built by assuming that the strain energy of an unsupported tensegrity structure is equivalent to the strain energy of a solid [39,40].

The strain energy of a tensegrity truss, according to the finite element method (FEM), is a quadratic form of nodal displacements **q**:

(1) $$E_{s}^{FEM}=\frac{1}{2}q^{T}\mathrm{Kq},$$

where $K=K_{L}+K_{G}$, $K_{L}$—global linear stiffness matrix, $K_{G}$—global geometric stiffness matrix. The self-stress of the structure is represented by the geometric stiffness matrix. The strain energy of a solid, according to the symmetric linear 3D elasticity theory (LTE), can be expressed as:

(2) $$E_{s}^{LTE}=\frac{1}{2}\int_{V}\varepsilon^{T}E\varepsilon \;dV,$$

where ε—vector of strain components (containing normal strains and shear strains), E—6 × 6 elasticity matrix in Voight’s notation [42] (including 21 independent coefficients). In order to analyse mechanical properties of the structure, such as Young’s moduli, shear moduli, Poisson’s ratios, etc., the strain energy of an unsupported tensegrity structure is compared to the strain energy of a cube of edge length *a*. It is assumed that the strain energy of the cube is constant in its whole volume. To compare the energies and to build the equivalent elasticity matrix, the nodal displacements **q** of the structure are expressed by the average mid-values of displacements and their derivatives in the centre of the cube of edge length *a*, with the use of Taylor series expansion. In case of small values of *a*, terms with the factor *a^n^* (*n* > 1) can be regarded as higher order terms and are omitted. This assumption leads, in a quantitative sense, to the infinitesimal model. Mechanical characteristics can be determined from the inverse elasticity matrix $H=E^{-1}$ [42]:

(3) $$H=[\begin{array}{cccccc} \frac{1}{E_{1}} & -\frac{V_{21}}{E_{2}} & -\frac{V_{31}}{E_{3}} & \frac{\lambda_{11}}{G_{1}} & \frac{\lambda_{21}}{G_{2}} & \frac{\lambda_{31}}{G_{3}}\\ -\frac{V_{12}}{E_{1}} & \frac{1}{E_{2}} & -\frac{V_{32}}{E_{3}} & \frac{\lambda_{12}}{G_{1}} & \frac{\lambda_{22}}{G_{2}} & \frac{\lambda_{32}}{G_{3}}\\ -\frac{V_{13}}{E_{1}} & -\frac{V_{23}}{E_{2}} & \frac{1}{E_{3}} & \frac{\lambda_{13}}{G_{1}} & \frac{\lambda_{23}}{G_{2}} & \frac{\lambda_{33}}{G_{3}}\\ \frac{\kappa_{11}}{E_{1}} & \frac{\kappa_{21}}{E_{2}} & \frac{\kappa_{31}}{E_{3}} & \frac{1}{G_{1}} & \frac{\mu_{21}}{G_{2}} & \frac{\mu_{31}}{G_{3}}\\ \frac{\kappa_{12}}{E_{1}} & \frac{\kappa_{22}}{E_{2}} & \frac{\kappa_{32}}{E_{3}} & \frac{\mu_{12}}{G_{1}} & \frac{1}{G_{2}} & \frac{\mu_{32}}{G_{3}}\\ \frac{\kappa_{13}}{E_{1}} & \frac{\kappa_{23}}{E_{2}} & \frac{\kappa_{33}}{E_{3}} & \frac{\mu_{13}}{G_{1}} & \frac{\mu_{23}}{G_{2}} & \frac{1}{G_{3}} \end{array}].$$

The following technical coefficients can be defined for anisotropic body: Young’s moduli (*E*), shear moduli (*G*), Poisson’s ratios (*ν*), coefficients (*μ*)—relations between shear strains in perpendicular directions, coefficients (*λ*)—relations between normal strains in three directions and shear strains in one direction, and coefficients (*κ*)—relations between shear strains in three directions and normal strains in one direction.

Moreover, limiting conditions for the values of mechanical characteristics can be found using the assumption that both matrices **E** and **H** have to be positive definite (see [44,45] for details).

The continuum analysis of tensegrities allows for: estimate the influence of self-stress on the behaviour of the system, assess how the characteristics of cables and struts affect the whole structure and determine the mechanical characteristics of materials or structures that are based on tensegrity modules.

In the continuum analysis of a unit cell, a 4-strut simplex module inscribed into a cube of edge length *a* (Figure 1) was considered. The module itself is an anisotropic structure, but as proved in the following sections it can be arranged in such a way that the material based on a simplex tensegrity pattern has orthotropic properties.

The elasticity matrix **E** obtained for the analysed module has the following form:

(4) $$E=\left[\begin{array}{cccccc} e_{11} & e_{12} & e_{13} & e_{14} & 0 & 0\\ & e_{11} & e_{13} & -e_{14} & 0 & 0\\ & & e_{33} & 0 & 0 & 0\\ & & & e_{12} & 0 & 0\\ & & & & e_{13} & 0\\ sym. & & & & & e_{13} \end{array}\right],$$

where:

$\begin{array}{l} e_{11}=\frac{2EA}{a^{2}}\left(0.314815+1.39827\cdot k-0.0794978\cdot \sigma \right),\\ e_{12}=\frac{EA}{a^{2}}\left(0.296296+0.707107\cdot k-0.0134742\cdot \sigma \right),\\ e_{13}=\frac{EA}{a^{2}}\left(0.740741+0.357771\cdot k+0.17247\cdot \sigma \right), \end{array}$
$\begin{array}{l} e_{14}=\frac{EA}{a^{2}}\left(-0.2222222-0.0808452\cdot \sigma \right),\\ e_{33}=\frac{2EA}{a^{2}}\left(0.592593+1.43108\cdot k-0.17247\cdot \sigma \right), \end{array}$
$k=\frac{{\left(EA\right)}_{cable}}{{\left(EA\right)}_{strut}}$, ${\left(EA\right)}_{strut}=EA$, $\sigma =\frac{S}{EA}$,
*E*—Young’s modulus of the strut, *A*—cross-sectional area of the strut.

Two characteristic parameters were used in the analysis: *k* and *σ*. Parameter *k* is defined as a ratio between the stiffness of cables and struts, parameter *σ* determines the level of self-stress.

It should be noticed that the proposed unit cell is an anisotropic structure, as there are non-zero coefficients $e_{14}$ and $e_{24}=e_{14}$ in the determined elasticity matrix (4). However, it is proved in the following sections that the material that is based on such unit cells exhibits orthotropic properties.

## 3. Tensegrity Cellular Material

Simplex tensegrity modules that are described in the previous section can be arranged in different patterns to form a material with certain properties. Depending on the type of the module used (with the basis rotated clockwise or counter clockwise) and the way in which the modules are connected, a material with different mechanical characteristics can be obtained. In the present paper, a material with orthotropic properties is proposed, as it exhibits some special features, such as a negative Poisson’s ratio.

**Figure 3:**
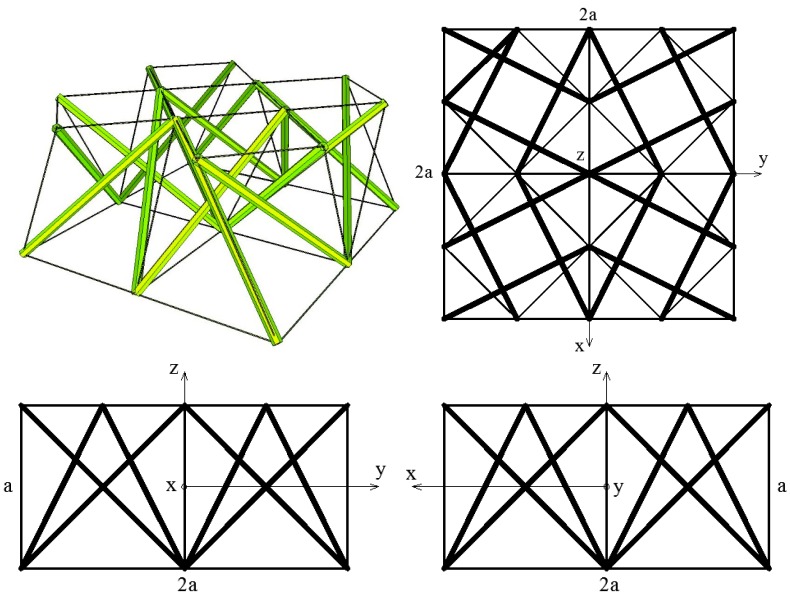
Geometry of a four-module layer—axonometry and three views.

Figure 3 presents a system consisting of four simplex modules that are connected through common cables of the lower bases and common nodes of the upper bases.

The following elasticity matrix **E** was determined for the analysed system using the continuum approach:

(5) $$E=\left[\begin{array}{cccccc} e_{11} & e_{12} & e_{13} & 0 & 0 & 0\\ & e_{11} & e_{13} & 0 & 0 & 0\\ & & e_{33} & 0 & 0 & 0\\ & & & e_{12} & 0 & 0\\ & & & & e_{13} & 0\\ sym. & & & & & e_{13} \end{array}\right],$$

where:

$\begin{array}{l} e_{11}=\frac{2EA}{a^{2}}\left(0.314815+1.13709\cdot k-0.0794978\cdot \sigma \right),\\ e_{12}=\frac{EA}{a^{2}}\left(0.296296+0.707107\cdot k-0.0134742\cdot \sigma \right),\\ e_{13}=\frac{EA}{a^{2}}\left(0.740741+0.268328\cdot k+0.17247\cdot \sigma \right),\\ e_{33}=\frac{2EA}{a^{2}}\left(0.592593+1.07331\cdot k-0.17247\cdot \sigma \right). \end{array}$

The obtained elasticity matrix (5) indicates that the four-module layer, although based on anisotropic unit cells, exhibits orthotropic properties.

**Figure 4:**
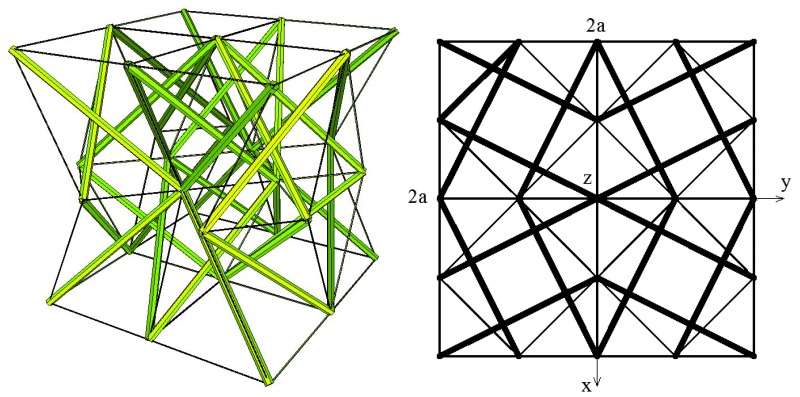

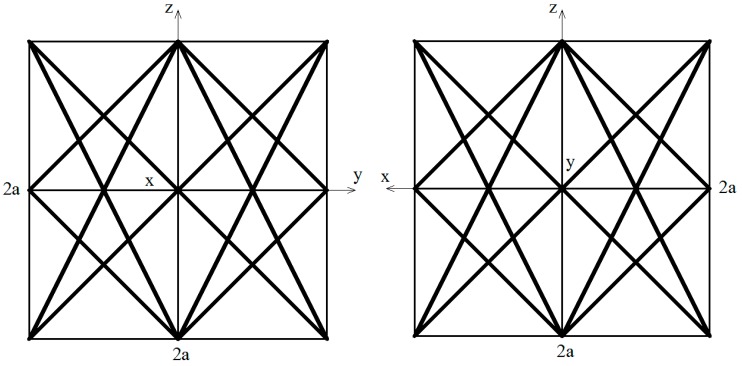
Geometry of an eight-module supercell—axonometry and three views.

Following this reasoning, an eight-module supercell (Figure 4), which was built from two four-module layers, was considered. The upper layer of the system was created by putting the four-module layer upside-down and connecting it with the bottom layer through common cables.

The elasticity matrix **E** that was obtained from the continuum analysis of the considered supercell has the following form:

(6) $$E=\left[\begin{array}{cccccc} e_{11} & e_{12} & e_{13} & 0 & 0 & 0\\ & e_{11} & e_{13} & 0 & 0 & 0\\ & & e_{33} & 0 & 0 & 0\\ & & & e_{12} & 0 & 0\\ & & & & e_{13} & 0\\ sym. & & & & & e_{13} \end{array}\right],$$

where:

$e_{11}=\frac{2EA}{a^{2}}\left(0.314815+0.960318\cdot k-0.0794978\cdot \sigma \right),$
$\begin{array}{l} e_{12}=\frac{EA}{a^{2}}\left(0.2962963+0.353553\cdot k-0.0134742\cdot \sigma \right),\\ e_{13}=\frac{EA}{a^{2}}\left(0.740741+0.268328\cdot k+0.17247\cdot \sigma \right),\\ e_{33}=\frac{2EA}{a^{2}}\left(0.592593+1.07331\cdot k-0.17247\cdot \sigma \right). \end{array}$

Similarly to the four-module layer, the analysed eight-module supercell has orthotropic properties.

In order to find mechanical characteristics of the structure, an inverse matrix $H=E^{-1}$ with seven independent coefficients was determined:

(7) $$H=E^{-1}=\left[\begin{array}{cccccc} \frac{1}{E_{1}} & -\frac{\nu_{12}}{E_{1}} & -\frac{\nu_{31}}{E_{3}} & 0 & 0 & 0\\ -\frac{\nu_{12}}{E_{1}} & \frac{1}{E_{1}} & -\frac{\nu_{31}}{E_{3}} & 0 & 0 & 0\\ -\frac{\nu_{13}}{E_{1}} & -\frac{\nu_{13}}{E_{1}} & \frac{1}{E_{3}} & 0 & 0 & 0\\ 0 & 0 & 0 & \frac{1}{G_{1}} & 0 & 0\\ 0 & 0 & 0 & 0 & \frac{1}{G_{2}} & 0\\ 0 & 0 & 0 & 0 & 0 & \frac{1}{G_{2}} \end{array}\right]$$

with the following values:

$\begin{array}{l} E_{1}=E_{2}=\frac{\left(e_{11}-e_{12}\right)\left(2e_{13}^{2}-\left(e_{11}+e_{12}\right)e_{33}\right)}{e_{13}^{2}-e_{11}e_{33}},\\ E_{3}=\frac{-2e_{13}^{2}+\left(e_{11}+e_{12}\right)e_{33}}{e_{11}+e_{12}},\\ G_{1}=e_{12},\\ G_{2}=G_{3}=e_{13}, \end{array}$
$\begin{array}{l} \nu_{12}=\nu_{21}=\frac{e_{13}^{2}-e_{12}e_{33}}{e_{13}^{2}-e_{11}e_{33}},\\ \nu_{13}=\nu_{23}=\frac{-e_{13}\left(e_{11}-e_{12}\right)}{e_{13}^{2}-e_{11}e_{33}},\\ \nu_{31}=\nu_{32}=\frac{e_{13}}{e_{11}+e_{12}}. \end{array}$

Symmetry of the matrix **H** generates the following condition: $\nu_{13}/E_{1}=\nu_{31}/E_{3}$. Moreover, the limiting conditions that are described in [44,45] resulting from the positive definition of the matrices **E** and **H** have to be satisfied: $E_{1}>0,\;E_{2}>0,\;E_{3}>0,$ $G_{1}>0,\;G_{2}>0,\;G_{3}>0,$ and $\nu_{12}\nu_{21}<1,\;\nu_{13}\nu_{31}<1,\;\nu_{23}\nu_{32}<1,$ $\nu_{12}\nu_{21}+\nu_{13}\nu_{31}+\nu_{23}\nu_{32}+\nu_{12}\nu_{31}\nu_{23}+\nu_{21}\nu_{13}\nu_{32}<1$.

Analysis of the above limiting conditions and the domains of the determined mechanical characteristics leads to conditions that limit the values of parameters *k* and *σ*. All of the considered conditions are described by curves, which, for engineering purposes, can be approximated with the equations of straight lines (see [46] for details). A general condition that is a common domain for all of the mechanical characteristics can be written, as follows:

(8) σ>3.69983 k.

**Figure 5:**
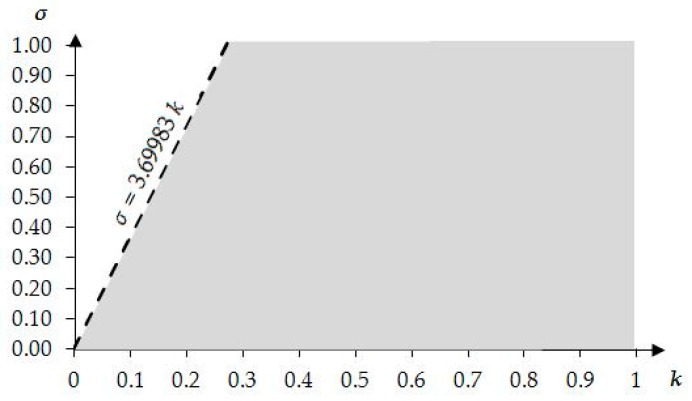
Range of possible values of the parameters *k* and *σ*.

In addition to the above condition, in the analyses presented in this paper the values of *k* and *σ* are limited to: k<1 and σ<1. Figure 5 presents a range of possible values of the considered parameters. The boundary line of the region σ>3.69983 k is also marked on the contour plots of the selected mechanical characteristics presented below.

**Figure 6:**
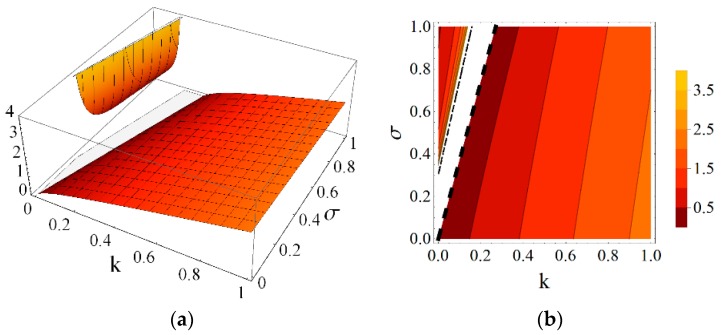
Young’s modulus $E_{1}$ (divided by the factor $EA/a^{2}$) with the limiting condition $E_{1}>0$: (**a**) three-dimensional (3D) plot; and (**b**) contour plot.

**Figure 7:**
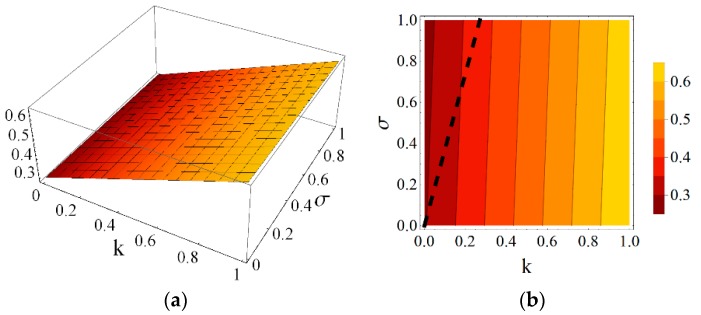
Shear modulus $G_{1}$ (divided by the factor $EA/a^{2}$) with the limiting condition $G_{1}>0$: (**a**) 3D plot; and (**b**) contour plot.

**Figure 8:**
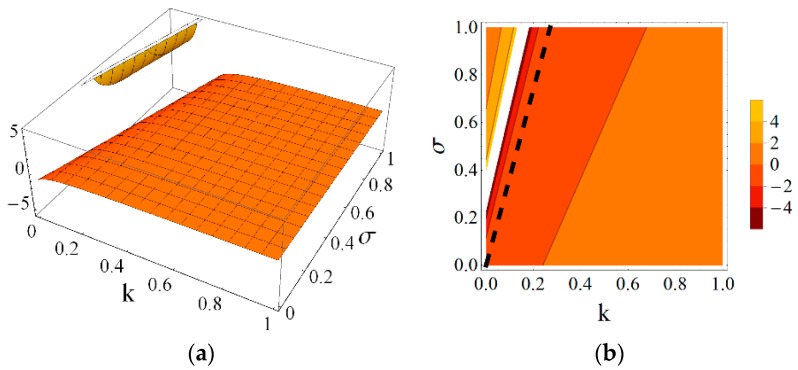
Poisson’s ratio $\nu_{12}=\nu_{21}$: (**a**) 3D plot; and, (**b**) contour plot.

**Figure 9:**
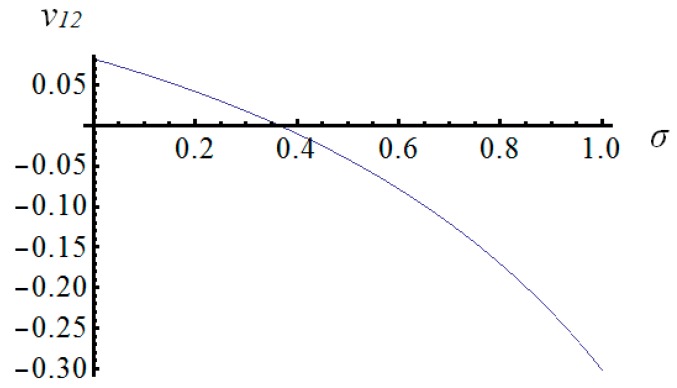
Poisson’s ratio $\nu_{12}=\nu_{21}$ changing sign (the plot for *k* = 0.4).

**Figure 10:**
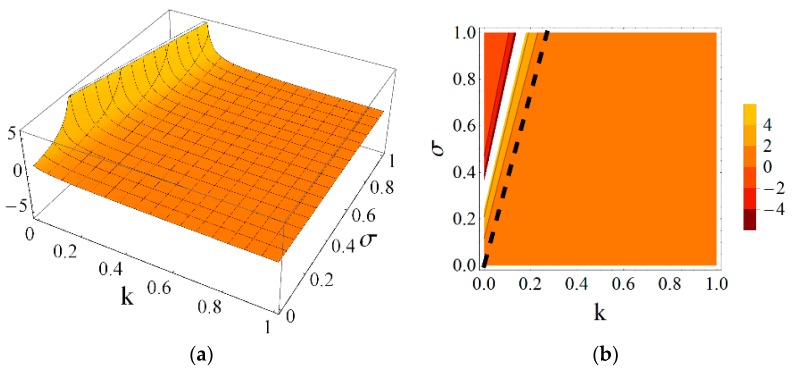
Poisson’s ratio $\nu_{13}=\nu_{23}$: (**a**) 3D plot; and (**b**) contour plot.

**Figure 11:**
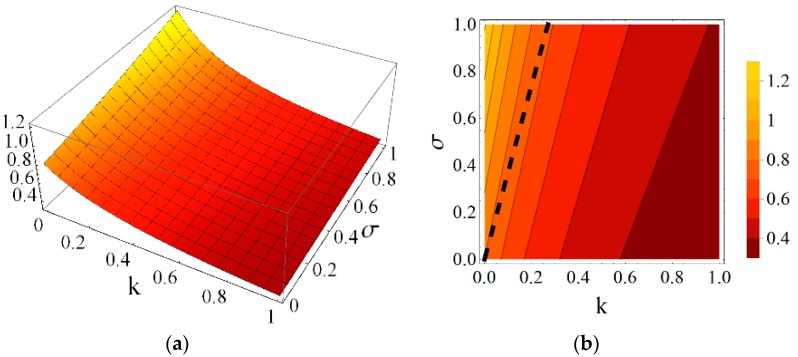
Poisson’s ratio $\nu_{31}=\nu_{32}$: (**a**) 3D plot; and (**b**) contour plot.

Using the results obtained from the presented continuum analysis, an influence of element properties and self-stress on the mechanical characteristics of the eight-module supercell can be determined. Figure 6, Figure 7, Figure 8, Figure 9, Figure 10 and Figure 11 show how the selected mechanical characteristics depend on the defined parameters *k* and *σ*. The values of these characteristics should be considered in the range that is shown in Figure 5. It should be noticed that all of the analysed mechanical characteristics are prone to structural control—their values might be controlled by adjusting either the properties of struts and cables or the values of prestressing forces in structural members. This feature distinguishes the proposed smart metamaterial from the traditional ones. Whereas, a typical material exhibits certain properties that are constant (assuming that the rheology is neglected), the characteristics of a smart material can be actively controlled. 

Parameter *k* depends on the physical and geometrical cable to strut ratio and can be fixed for the supercell at a certain level. Slightly different is the role of self-stress parameter *σ,* which can be adjusted during exploitation of the material to control the values of elastic coefficients. As it is seen in Figure 6 and Figure 7, the influence of these parameters on the Young’s and shear modulus is quite significant.

The most important features that characterize mechanical metamaterials are negative values of Poisson’s ratio. In the case of the proposed eight-module supercell Poisson’s ratio $\nu_{12}$ (and $\nu_{21}=\nu_{12}$), can reach negative as well as positive values in the considered range of the parameters *k* and *σ* (Figure 8 and Figure 9, as an example for the fixed value of the parameter *k* = 0.4). The influence of the self-stress parameter *σ* on the value of Poisson’s ratio $\nu_{12}$ is significant. The gaps between the positive and negative values of the Poisson’s ratios $\nu_{12}$ and $\nu_{13}$ visible in Figure 8 and Figure 10 result from the domains of these functions. However, they are not taken into account as they lie outside the considered range (Figure 5).

The other Poisson’s ratios $\nu_{13}$ and $\nu_{31}$ are always positive (Figure 10 and Figure 11) in the considered domain (Figure 5). While the coefficient $\nu_{13}$ is not very sensitive to *k* and *σ*, the other one $\nu_{31}$ can be adjusted by changing these two parameters.

It should be highlighted that in the case of the analysed system, the selected Poisson’s ratios can not only have negative values, but they can also change sign (Figure 9). It means that the proposed metamaterial behaves differently depending on the adopted parameters *k* and *σ*. This is a unique feature of smart metamaterials. Such a material can act like a standard material with positive Poisson’s ratios, and, in the same time, it can be changed into a metamaterial with negative values of these mechanical characteristics.

**Figure 12:**
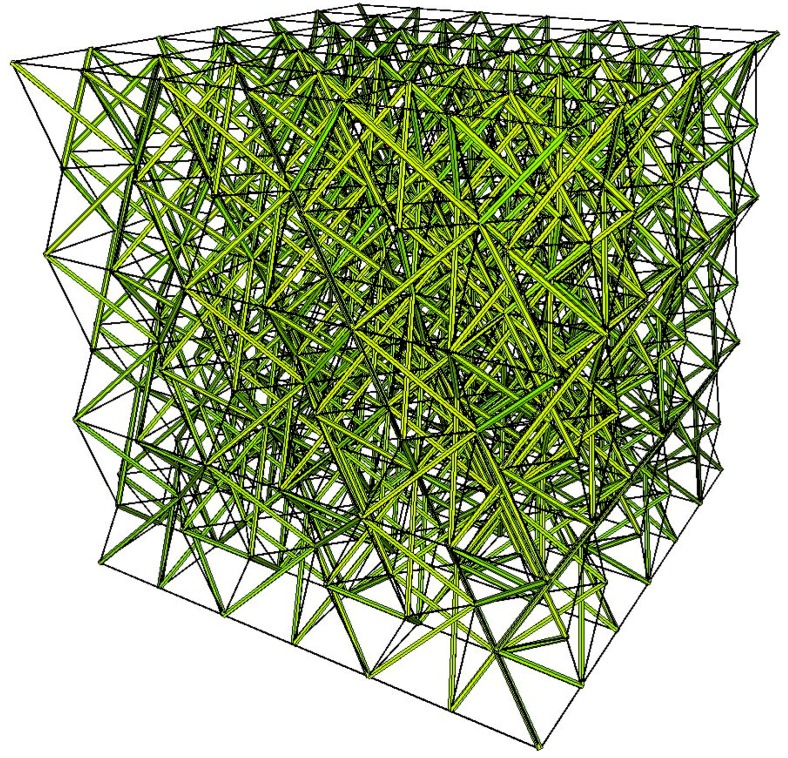
Metamaterial built from the eight-module supercells.

The considered eight-module supercells can be used to build a metamaterial of any volume. The properties of such a material are the same as the properties of the supercell. An example of a metamaterial that is built from the analysed eight-module supercells is presented in Figure 12.

## 4. Conclusions

The present paper focuses on the development and analysis of a novel cellular metamaterial based on the simplex tensegrity pattern. The proposed material is constructed from supercells, each of which consists of eight 4-strut simplex modules. Using continuum model different structures are analyzed: a unit cell, a four-module layer, and an eight-module supercell. The continuum analysis of tensegrities allows for: estimating the influence of self-stress on the behaviour of the system, asses how the characteristics of cables and struts affect the whole structure and determine mechanical characteristics of materials or structures based on tensegrity patterns.

The proposed unit cell is an anisotropic structure. However, it can be arranged in such a way that that the material based on the simplex tensegrity pattern exhibits orthotropic properties. Such a material is considered in this paper. The developed metamaterial has some unusual properties, which are typical for smart structures. It is possible to control its mechanical characteristics by adjusting the level of self-stress or by changing the properties of structural members.

One of the most important features of the proposed cellular metamaterial is a unique behaviour of some of its Poisson’s ratios. Depending on the applied control parameters, they can be either positive or negative. This feature is of a great importance as far as the active control of the system is concerned.

Moreover, the proposed metamaterial exhibits features which characterize smart systems. Its mechanical properties, such as Young’s moduli, shear moduli and Poisson’s ratios, can be adjusted and controlled during the exploitation via the self-stress parameter *σ* to satisfy the self-control, self-diagnosis, active-control, as well as self-repair conditions (see [20,37,46] for details of smart tensegrity structures). It is possible due to the occurrence of infinitesimal mechanisms that are balanced with self-stress states.

The results that are presented in this paper indicate a great potential that lies in the proposed metamaterial. Thanks to its unique features, such as the negative Poisson’s ratio, controllability, sensitivity to the self-stress, the material can be applied in smart structural systems. The members constructed from such a material can be designed to adapt to a functional need by modifying their shape, stiffness, or damping characteristics in order to minimize the deflection and possible damage.

In the future works, it is worth developing and analysing smart metamaterials based on different tensegrity patterns. Other modules are to be considered as unit cells in various arrangements—orthotropic and anisotropic ones. The proposed systems are to be considered from the point of view mechanical properties (using the continuum approach), as well as inherent smartness and the conditions that have to be satisfied in order to regard a structure as smart.

## References

[1] Cui T.J., Smith D.R., Liu R. (2010). Metamaterials. Theory, Design and Applications.

[2] Engheta N., Ziolkowski R.W. (2006). Metamaterials. Physics and Engineering Explorations.

[3] Singh G., Ni R., Marwaha A. (2015). A review of metamaterials and its applications. Int. J. Eng. Trends Technol..

[4] Schurig D., Mock J.J., Justice B.J., Cummer S.A., Pendry J.B., Starr A.F., Smith D.R. (2006). Metamaterial electromagnetic cloak at microwave frequencies. Science.

[5] Soukoulis C.M., Linden S., Wegener M. (2007). Negative refractive index at optical wavelengths. Science.

[6] Soukoulis C.M., Wegener M. (2011). Past achievements and future challenges in the development of three-dimensional photonic metamaterials. Natl. Photonics.

[7] Vora A., Gwamuri J., Pala N., Kulkarni A., Pearce J.M., Guney D.O. (2014). Exchenging ohmic losses in metamaterial absorbers with useful optical absorption for photovoltaics. Sci. Rep..

[8] Wu C., Neuner B., John J., Milder A., Zollars B., Savoy S., Shvets G. (2012). Metamaterial-based integrated plasmonic absorber/emitter for solar thermos-photovoltaic systems. J. Opt..

[9] Brule S., Javelaud E.H., Enoch S., Guenneau S. (2014). Experiments on seismic metamaterials: Molding surface waves. Phys. Rev. Lett..

[10] Chen H., Chan C.T. (2007). Acoustic cloaking in three dimensions using acoustic metamaterials. Appl. Phys. Lett..

[11] Mei J., Ma G., Yang M., Yang Z., Wen W., Sheng P. (2012). Dark acoustic metamaterials as super absorbers for low-frequency sound. Nat. Commun..

[12] Lee J.B., Peng S., Yang D., Roh Y.H., Funabashi H., Park N., Rice E.J., Chen L., Long R., Wu M. (2012). A mechanical metamaterial made from a DNA hydrogel. Nat. Nanotechnol..

[13] Bertoldi K., Reis P.M., Willshaw S., Mullin T. (2010). Negative poisson’s ratio behavior by an elastic instability. Adv. Mater..

[14] Kadic M., Buckmann T., Stenger N., Thiel M. (2012). On the practicability of pentamode mechanical metamaterials. Appl. Phys. Lett..

[15] Lee J.H., Singer J.P., Thomas E.L. (2012). Micro-/Nanostructured mechanical metamaterials. Adv. Mater..

[16] Zheng X., Lee H., Weisgraber T.H., Shusteff M., Deotte J., Duoss E.B., Kuntz J.D., Biener M.M., Ge Q., Jackson J.A. (2014). Ultra-light, ultra-stiff mechanical metamaterials. Science.

[17] Farshad M. (1995). Intelligent materials and structures. Sci. Iran..

[18] Lopes Junior V., Steffen V., Savi M.A. (2016). Dynamics of Smart Systems and Structures.

[19] Schwartz M. (2009). Smart Materials.

[20] Gilewski W., Al Sabouni-Zawadzka A. (2015). On possible applications of smart structures controlled by self-stress. Arch. Civ. Mech. Eng..

[21] Schenk M., Guest S.D. (2013). Geometry of Miura-folded metamaterials. Proc. Natl. Acad. Sci. USA.

[22] Lv C., Krishnaraju D., Konjevod G., Yu H., Jiand H. (2014). Origami based mechanical metamaterials. Sci. Rep..

[23] Silverberg J.L., Evans A.A., McLeod L., Hayward R.C., Hull T., Santangelo C.D., Cohen I. (2014). Using origami design principles to fold reprogrammable mechanical metamaterials. Science.

[24] Eidini M., Paulino G.H. (2015). Unraveling metamaterial properties in zigzag-base folded sheets. Sci. Adv..

[25] Filipov E.T., Tachi T., Paulino G.H. (2015). Origami tubes assembled into stiff, yet reconfigurable structures and metamaterials. Proc. Natl. Acad. Sci. USA.

[26] Waitukaitis S., Menaut R., Chen B.G.G., van Hecke M. (2015). Origami multistability: from single vertices to metasheets. Phys. Rev. Lett..

[27] Skelton R.E., de Oliveira M.C. (2009). Tensegrity Systems.

[28] Motro R. (2003). Tensegrity: Structural Systems for the Future.

[29] Wroldsen A.S. (2007). Modelling and Control of Tensegrity Structures. Ph.D. Thesis.

[30] Calladine C.R., Pellegrino S. (1991). First-order infinitesimal mechanisms. Int. J. Solids Struct..

[31] Adam B., Smith I.F.C. (2006). Learning, self-diagnosis and multi-objective control of an active tensegrity structure. Adv. Eng. Struct. Mech. Constr..

[32] Liu K., Wu J., Paulino G.H., Qi H.J. (2017). Programable deployment of tensegrity structures by stimulus-responsive polymers. Sci. Rep..

[33] Adam B., Smith I.F.C. (2007). Self-diagnosis and self-repair of an active tensegrity structure. J. Struct. Eng..

[34] Bel Hadj Ali N., Smith I.F.C. (2010). Dynamic behavior and vibration control of a tensegrity structure. Int. J. Solids Struct..

[35] Fest E., Shea K., Smith I.F.C. (2004). Active tensegrity structure. J. Struct. Eng..

[36] Moored K.W., Kemp T.H., Houle N.E., Bart-Smith H. (2011). Analytical predictions, optimization, and design of a tensegrity-based artificial pectorial fin. Int. J. Space Struct..

[37] Al Sabouni-Zawadzka A., Gilewski W. Inherent smartness of tensegrity structures—Structural elements applications. Proceedings of the International Association for Shell and Spatial Structures (IASS).

[38] Koohestani K., Guest S.D. (2013). A new approach to the analytical and numerical form-finding of tensegrity structures. Int. J. Solids Struct..

[39] Gilewski W., Al Sabouni-Zawadzka A. (2018). Continuum model of cable-strut structures with self-stress included. Int. J. Space Struct..

[40] Gilewski W., Kasprzak A., Szcześniak W., Ataman M. (2013). Description of the influence of self-stress on the properties of tensegrity modules using a continuum approach. Theroretical Foundations of Civil Engineering, Vol. IV. Technical Mechanics.

[41] Rimoli J.J., Pal R.K. (2017). Mechanical response of 3-dimensional tensegrity lattices. Compos. Part B.

[42] Green A.E., Zerna W. (1968). Theoretical Elasticity.

[43] Chadwick P., Vianello M., Cowin S. (2001). A new proof that the number of linear elastic symmetries is eight. J. Mech. Phys. Solids.

[44] Ting T.C.T. (1996). Positive definiteness of anisotropic elastic constants. Math. Mech. Sloids.

[45] Zheng Q.S., Chen T. (2001). New perspective on Poisson’s ratio of elastic solids. Acta Mech..

[46] Al Sabouni-Zawadzka A. (2016). On Possible Applications of Smart Structures in Bridge Engineering. Ph.D. Thesis.

